# Molecular Prevalence and Identification of Zoonotic *Plasmodium* spp., Including *Plasmodium knowlesi*, *Plasmodium cynomolgi*, and *Plasmodium inui*, in Long-Tailed Macaques (*Macaca fascicularis*) of Southern Thailand

**DOI:** 10.1155/vmi/3024193

**Published:** 2025-06-23

**Authors:** Thanawat Hmaidee, Rucksak Rucksaken, Supakarn Kaewchot, Piya Sereerak, Salintorn Thongsahuan, Thitichai Jarudecha, Sakulchit Wichainchot, Phakorn Wilaisri, Chanapath Thabthimsri, Perm Premphoolsawat, Wanat Sricharern

**Affiliations:** ^1^Department of Veterinary Nursing, Faculty of Veterinary Technology, Kasetsart University, Bangkok, Thailand; ^2^Royal Thai Government Department of National Park Wildlife and Plant Conservation, Bangkok, Thailand; ^3^Department of Veterinary Technology, Faculty of Veterinary Technology, Kasetsart University, Bangkok, Thailand; ^4^Department of Parasitology, Faculty of Medicine, Kasetsart University, Bangkok, Thailand

**Keywords:** long-tailed macaque, *Macaca fascicularis*, malaria, *Plasmodium* spp., Thailand

## Abstract

Zoonotic malaria, caused by simian *Plasmodium* spp., poses a major public health challenge in Southeast Asia, including Thailand, where long-tailed macaques (*Macaca fascicularis*) serve as natural reservoirs. This study investigated the molecular prevalence and species identification of zoonotic simian *Plasmodium* spp. in macaques from four provinces in Southern Thailand: Phetchaburi, Satun, Phang Nga, and Surat Thani. A total of 310 blood samples were collected between May 2023 and June 2024 and analyzed using nested and seminested polymerase chain reaction (PCR) techniques targeting the 18S rRNA gene. Sequencing analyses confirmed the presence of zoonotic *Plasmodium* species. Overall, 11.3% (35/310; 95% CI: 7.9–15.3) of the macaques tested positive, with *Plasmodium inui* being the most prevalent species at 9.4% (29/310), followed by *Plasmodium knowlesi* and *Plasmodium cynomolgi*, each at 0.9% (3/310). The highest prevalence was observed in Surat Thani at 18% (18/100). These findings underscore the zoonotic potential of simian malaria and its geographic distribution in Southern Thailand, which may be associated with the significant increase in macaque populations and their expanding habitat overlap with human communities. In conclusion, this study highlights the major role of long-tailed macaques as reservoirs for zoonotic *Plasmodium* spp. Enhanced surveillance and community awareness are crucial for mitigating cross-species transmission and improving malaria control.

## 1. Introduction

Malaria is a life-threatening infectious disease caused by protozoan parasites of the *Plasmodium* genus, primarily transmitted through the bites of infected female *Anopheles* mosquitoes [1]. The disease remains endemic in tropical and subtropical regions, including Africa, Central and South America, Asia, and Oceania, where environmental conditions, such as temperature and rainfall, support favorable habitats for *Anopheles* vectors [2]. According to the World Health Organization report in 2024, a total of approximately 263 million cases of malaria were reported globally, with an estimated 597,000 malaria deaths worldwide in the year 2023 [3]. While malaria transmission has traditionally been less prevalent in urban areas, rapid urbanization and population growth have facilitated the movement of infected individuals, contributing to the spread of malaria pathogens across both urban and rural environments [2, 4]. The life cycle of *Plasmodium* spp. is complex, requiring both vertebrate and invertebrate (mosquitoes) hosts. In humans, the incubation period of the parasite typically ranges from 3 to 14 days, during which the infection remains asymptomatic. Once the parasites mature and invade red blood cells, affected individuals develop clinical symptoms, such as fever, chills, headache, muscle aches, and fatigue, which are characteristic of malaria [5].

Five *Plasmodium* species are known to cause malaria in humans: *Plasmodium falciparum*, *Plasmodium vivax*, *Plasmodium malariae*, *Plasmodium ovale*, and *Plasmodium knowlesi* [6]. However, malaria is not restricted to humans. Various *Plasmodium* species also infect a wide range of animal hosts. Avian hosts harbor species, such as *Plasmodium relictum*, *Plasmodium elongatum*, *Plasmodium circumflexum*, *Plasmodium matutinum*, and *Plasmodium vaughani* [7–9], while reptilian hosts carry *Plasmodium floridense* and *Plasmodium colombiense* [6, 10, 11]. Rodent hosts are infected by species, including *Plasmodium yoelii*, *Plasmodium berghei*, *Plasmodium chabaudi*, and *Plasmodium vinckei* [12]. Notably, *Plasmodium* spp. infecting nonhuman primates pose a major zoonotic threat, as evidenced by documented cases of cross-species transmission, particularly from macaques to humans. Such instances complicated malaria control efforts in regions where human and wildlife populations coexist [13].

Nonhuman primates, particularly macaques, play an important role in zoonotic disease transmission. Several species of macaques are found in Thailand, consisting of the long-tailed macaque (*Macaca fascicularis*), pig-tailed macaque (*Macaca nemestrina*), stump-tailed macaque (*Macaca arctoides*), Assam macaque (*Macaca assamensis*), Indochinese rhesus macaque (*Macaca mulatta*), and northern pig-tailed macaque (*Macaca leonina*) [14, 15]. Frequently, these macaques inhabit areas near human settlements, leading to increased human–wildlife interactions. Such close contact has raised concerns regarding the transmission of zoonotic pathogens from macaques to humans. For example, studies have documented the presence of several potentially zoonotic pathogens in macaques in Thailand, including the herpes B virus, which has a high mortality rate in humans [16, 17], as well as *Bartonella quintana* [18], *Giardia duodenalis* and *Cryptosporidium* sp. [19], and *Trichuris trichiura* and *Hymenolepis diminuta* [20]. In addition, macaques serve as natural hosts for several *Plasmodium* species, consisting of *P. knowlesi*, *Plasmodium cynomolgi*, *Plasmodium inui*, *Plasmodium coatneyi*, and *Plasmodium fieldi* [14, 21]. The transmission of these parasites from macaques to humans, particularly in regions where macaques reside near residential areas, poses major public health concern. Several provinces in Thailand, such as Tak, Ranong, Yala, Narathiwat, Prachuap Khiri Khan, and Chanthaburi, have reported human infections with *P. knowlesi* and *P. cynomolgi* [22]. In addition, another study has reported cases of *P. knowlesi* infection in humans in Surat Thani province [23].

Despite growing evidence of zoonotic malaria in Thailand, there is still a lack of research on the transmission of *Plasmodium* species between humans and macaques. Although some studies have identified *P. knowlesi* and *P. cynomolgi* in macaque populations [24–26], there remains a pressing need for more comprehensive research on the prevalence and distribution of *Plasmodium* species in macaques across different areas of the country. Thus, the current study aimed to investigate the molecular prevalence of *Plasmodium* spp. infections in long-tailed macaques from Southern Thailand and to identify specific *Plasmodium* species using molecular techniques.

## 2. Materials and Methods

### 2.1. Ethical Approval

The protocols involving animal use in this research were approved by the Kasetsart University Institutional Animal Care and Use Committee (IACUC), Bangkok, Thailand, under the Ethical Review Board of the Office of the National Research Council of Thailand (NRCT) (Approval ID: ACKU67-VTN-001). This approval ensured the ethical conduct of the scientific research.

### 2.2. Study Period and Locations

**Figure 1:**
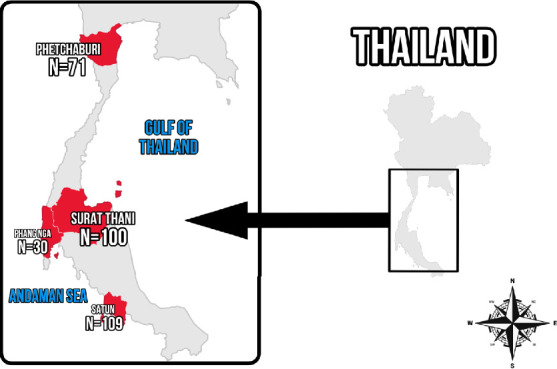
Map of Southern Thailand showing sampling sites for long-tailed macaques in Phetchaburi, Surat Thani, Phang Nga, and Satun provinces (modified from https://www.pikpng.com/transpng/iRixJiR/).

Blood samples were collected from May 2023 to June 2024 from long-tailed macaques in four provinces of Southern Thailand: Phetchaburi, Phang Nga, Surat Thani, and Satun (Figure 1). Laboratory analyses were conducted at the Faculty of Veterinary Technology, Kasetsart University, Bangkok, Thailand.

### 2.3. Sample Collection

The sample size was estimated using Epitools (https://epitools.ausvet.com.au) with an expected proportion of 0.29 based on another study in Thailand [14]. The precision of the estimate and the confidence level were set as 0.05% and 95%, respectively. In total, 310 blood samples were collected from free-ranging long-tailed macaques in the study areas, comprising 71 samples (37 males and 34 females) from Ban Laem district, Phetchaburi province; 109 samples (61 males and 48 females) from Mueang Satun district, Satun province; 30 samples (14 males 16 females) from Mueang Phang Nga district, Phang Nga province; and 100 samples (68 males and 32 females) from Phunphin district, Surat Thani province. The macaques were humanely captured and initially sedated with xylazine hydrochloride (0.5–2 mg/kg body weight), followed by anesthesia with tiletamine-zolazepam (2–5 mg/kg body weight) [14]. Both drugs were administered via intramuscular injection, in accordance with the approved protocol. Approximately 1 mL of blood was collected from the femoral vein of each individual into tubes containing ethylenediaminetetraacetic acid (EDTA). All procedures were performed by licensed veterinarians from the Department of National Parks, Wildlife and Plant Conservation. The samples were kept cool during transportation to the laboratory and aliquoted into 200 μL portions in 1.5 mL tubes for storage at −40°C prior to DNA extraction.

### 2.4. Molecular Analysis

DNA was extracted from 200 μL of blood using a QIAamp® DNA Blood Mini Kit (QIAGEN; Hilden, Germany), following the manufacturer's instructions. Extracted DNA was stored at −40°C until further analysis. The detection of *Plasmodium* spp. was conducted using nested polymerase chain reaction (PCR) targeting the partial 18S rRNA gene. The genus-specific primers from Singh et al. [27] were used for the amplification. The primary PCR utilized the primers rPLU6 and rPLU5 under the following thermal cycling conditions: 95°C for 5 min; 35 cycles of 95°C for 30 s, 55°C for 60 s, and 72°C for 60 s; followed by a final extension at 72°C for 10 min. The secondary PCR used the primers rPLU3 and rPLU4 with the same thermal cycling profile as above. The expected amplicon sizes were 1100 bp for the primary PCR and 240 bp for the nested PCR. The 20 μL reaction mixture consisted of 10X buffer, 50 mM MgCl_2_, 10 mM dNTPs, 10 μM of each primer, and 5 U/μL of Taq polymerase (Invitrogen; Waltham, MA, USA).

Since the initial nested PCR method produced relatively low detection rates, a seminested PCR protocol, as reported by Imwong et al., was used to confirm the results [28]. This genus-specific primer set targeted the 18S rRNA gene of multiple simian *Plasmodium* species, which consisted of *P. cynomolgi*, *P. knowlesi*, *P. inui*, *P. coatneyi*, *P. fieldi*, *P. chabaudi*, *P. berghei*, *P. brasilianum*, *P. simium*, *P. semiovale*, *P. fragile*, *P. vinckei*, *P. yoelii*, and *P. adleria*. The primary PCR utilized the PlasmoM_N1F and PlasmoM_N1R primers under the following thermal cycling conditions: initial denaturation at 94°C for 3 min; 30 cycles of 94°C for 60 s, 53°C for 60 s, and 72°C for 60 s; followed by a final extension at 72°C for 10 min. Secondary PCR used the primers PlasmoM_N2F and PlasmoM_N1R with identical cycling conditions to those immediately above. The expected amplicon sizes ranged from 233 to 298 bp. The 20 μL reaction mixture was prepared using the same conditions as those described above for the nested PCR method. A summary of the primers used in the current study is provided in Table 1.

### 2.5. DNA Sequencing Analysis

The PCR products were analyzed using 1.2% agarose gel electrophoresis with Tris-acetate-EDTA buffer and visualized under UV transillumination after nucleic acid staining with Thermo Scientific™ 6X DNA gel loading dye (Thermo Fisher Scientific; Waltham, MA, USA). Due to the similarity in amplicon sizes across different *Plasmodium* species, species-level identification was not performed based on gel electrophoresis results. Positive samples of the expected size for *Plasmodium* spp. were purified using a GenepHlow™ Gel/PCR Kit (Geneaid Biotech Ltd.; New Taipei City, Taiwan). Purified DNA was sequenced using the Sanger methods (ATGC Company Limited; Pathum Thani, Thailand).

### 2.6. Statistical Analysis

Statistical analyses were conducted using the STATA software Version 15.1 (Stata Corporation; Texas, USA). The odds ratio (OR) with the corresponding 95% confidence interval (95% CI) and *p* value were calculated to assess the associations between *Plasmodium* spp. infection and variable characteristics such as host sex and sampling location. A multivariable logistic regression model was used for the analysis. Statistical significance was defined as *p* < 0.05.

## 3. Results

From a total of 310 blood samples collected from long-tailed macaques in Southern Thailand, *Plasmodium* spp. infections were detected using nested and seminested PCR methods. Based on the analysis, 11.3% of the samples (35/310; 95% CI: 8.0–15.3) tested positive for *Plasmodium* spp. The identified species consisted of *P. inui* at 9.3% (29/310; 95% CI: 6.3–13.2), *P. knowlesi* at 0.9% (3/310; 95% CI: 0.2–2.8), and *P. cynomolgi* at 0.9% (3/310; 95% CI: 0.2–2.8). Focusing on the male macaques (*n* = 180), the prevalence of *P. inui* was 11.7% (21/180; 95% CI: 7.4–17.3), and that of *P. cynomolgi* was 1.7% (3/180; 95% CI: 0.3–4.8), with no detection of *P. knowlesi*. This resulted in an overall *Plasmodium* spp. prevalence of 13.3% (24/180; 95% CI: 8.7–19.2) among male macaques, which was higher than the prevalence observed in females. Among the female macaques (*n* = 130), the overall prevalence was 8.5% (11/130; 95% CI: 4.3–14.6), with *P. inui* detected in 6.1% (8/130; 95% CI: 2.7–11.8) and *P. knowlesi* in 2.3% (3/130; 95% CI: 0.5–6.6), while there were no cases of *P. cynomolgi* (Table 2).

The prevalence of *Plasmodium* spp. infections in the long-tailed macaques varied across the studied locations. The highest prevalence was observed in Phunphin district, Surat Thani province, at 18% (18/100; 95% CI: 11.0–26.9), with *P. inui* detected in 13 cases, *P. cynomolgi* in 2 cases, and *P. knowlesi* in 3 cases. Notably, Surat Thani was the only province where all three species were identified. The second highest prevalence was in Mueang Satun district, Satun province, at 13.8% (15/109; 95% CI: 7.9–21.7), consisting of 14 cases of *P. inui* and 1 case of *P. cynomolgi*. In Mueang Phang Nga district, Phang Nga province, the prevalence was 3.3% (1/30; 95% CI: 0.1–17.2), with *P. inui* being the sole species detected. Similarly, Ban Laem district, Phetchaburi province, had a prevalence of 1.4% (1/71; 95% CI: 0–7.6), also limited to *P. inui*. These findings indicated that *P. inui* was the only species present at all the studied locations. The nucleotide sequences of the 18S rRNA gene for all *Plasmodium* spp. detected in the long-tailed macaques in Thailand during this study were submitted to GenBank under the accession numbers PQ559856–PQ559867 and PQ678665–PQ678691 (Table 3).

Based on the nucleotide sequence analysis of the partial 18S rRNA sequences, *P. inui* displayed 99.48%–100% identity with sequences from *P. inui* found in *M. fascicularis* in Malaysia and wild macaques in Thailand (GenBank accession numbers: FJ619093, FJ619095, EU400391, and EU400397). *P. knowlesi* displayed 98.06% homology with *P. knowlesi* from *M. fascicularis* in Malaysia (KT852930), while *P. cynomolgi* displayed 98.53% identity with *P. cynomolgi* from Japan (AB2872989 and AB287290). The nucleotide sequences of the 18S rRNA gene for all *Plasmodium* spp. detected in the long-tailed macaques in Thailand during this study were submitted to GenBank under the accession numbers PQ559856–PQ559867 and PQ678665–PQ678691 (Table 3).

A comprehensive statistical analysis was conducted to evaluate the relationships between *Plasmodium* spp. infection and either the sex or geographical location of the long-tailed macaques. Based on the results, there was no significant association between *Plasmodium* spp. infection and the sex of the macaques after adjustment by location. In contrast, geographical location emerged as a critical determinant of infection risk. Specifically, the adjusted ORs revealed that macaques residing in Satun and Surat Thani provinces were 11.06 times and 14.61 times more likely, respectively, to be infected with *Plasmodium* spp. than macaques in Phetchaburi province after adjustment by sex (Table 4).

## 4. Discussion

The current study identified three species of simian *Plasmodium* spp. (*P. inui*, *P. knowlesi*, and *P. cynomolgi*) in wild long-tailed macaques from four provinces in Southern Thailand. To the best of our knowledge, this was the first report of these parasites in certain areas based on molecular methods, specifically *P. inui*, *P. knowlesi*, and *P. cynomolgi* in Surat Thani province, as well as *P. inui* in Phetchaburi province. These findings were consistent with other studies conducted in Malaysia, the Philippines, Vietnam, Myanmar, Cambodia, and Laos that reported these three *Plasmodium* spp. in *M. fascicularis* populations [29–32]. The detection of *P. knowlesi* in macaques in Surat Thani was particularly important, as it was consistent with previous reports of *P. knowlesi* infections in humans from the same province [23]. This finding suggested a potential zoonotic transmission of *P. knowlesi* from macaques to humans. Furthermore, *Plasmodium* spp. infections have been documented in long-tailed macaques in Thailand, including *P. cynomolgi* and *P. inui* in Saraburi and Ratchaburi provinces in Central Thailand [31]. In Southern Thailand*, P. inui* was identified in Ranong and Krabi provinces, while both *P. inui* and *P. cynomolgi* were reported in Narathiwat and Songkhla provinces [17, 26, 31].

In addition to these three species, other simian *Plasmodium* spp. have been detected in long-tailed macaques in Thailand, specifically *P. coatneyi* [31] and *P. fieldi* [26, 31]. Furthermore, in addition to long-tailed macaques, diverse *Plasmodium* species have been identified in other macaque species. For example, *P. fieldi* and *P. coatneyi* have been reported in stump-tailed macaques [14], while *P. knowlesi* was reported in pig-tailed macaques [24]. These findings suggested that the variety of macaque species in Thailand served as major reservoir hosts, potentially contributing to the transmission of *Plasmodium* spp. among macaques and, importantly, to humans.

The prevalence of *Plasmodium* infection in the current study was 11.3% (35/310), which was lower than that in other studies. For example, a prevalence of 13% (100/772) was reported in long-tailed macaques in Thailand in 2024 [31], 29% (23/93) in stump-tailed macaques in 2020 [14], and 64.1% (177/276) in long-tailed macaques across Southeast Asia, including Laos, Singapore, Cambodia, the Philippines, and Indonesia, in 2016 [32]. Among the *Plasmodium* species identified in the current study, *P. inui* was the most prevalent, accounting for 9.4% (29/310), followed by *P. cynomolgi* and *P. knowlesi*, each representing 0.9% (3/310). These findings aligned with other research conducted in Southern Thailand [26] and in various regions of Thailand, including the eastern, western, southern, and northern areas [14], that also identified *P. inui* as the dominant species. Similarly, studies from Malaysia in 2011 and 2015 highlighted the high prevalence and parasitemia rates of *P. inui* [33]. However, other research reported contrasting findings, with a higher prevalence of *P. cynomolgi* compared to *P. inui* [31, 32].

A key limitation of this study is the absence of blood film microscopy (BFMP), which is traditionally used alongside molecular methods for *Plasmodium* diagnosis. This was due to practical constraints during fieldwork, including limited time for immediate blood smear preparation and sample transport logistics [34]. However, microscopy-based diagnosis has its own challenges, as it requires a high level of expertise to accurately differentiate *Plasmodium* species. Misidentification can occur, particularly when morphologically similar species are present [35]. Additionally, microscopy has lower sensitivity than molecular methods, especially when parasite densities are low [35].

Despite the absence of microscopy, this study employed PCR-based detection and sequencing, which are highly specific and sensitive, allowing for accurate species identification [35]. While some studies have integrated both techniques [36], others have relied exclusively on molecular methods [14, 28]. Therefore, despite this limitation, our findings remain robust and contribute valuable insights into the prevalence of simian malaria in the study region. Future studies that integrate both techniques may further enhance diagnostic accuracy and comparative analysis. Another limitation of this study was the use of genus-specific primers (PlasmoM_N2F and PlasmoM_N1R), which were effective in detecting *Plasmodium* DNA but could not reliably distinguish between species based on amplicon size, particularly in cases of mixed infections. The absence of species-specific primers, such as those described by Lee et al. [37], restricted the ability to accurately and quickly identify individual *Plasmodium* species. Future research incorporating species-specific primers would improve detection accuracy and offer a clearer understanding of species diversity.

In the current study, the male macaques had a higher infection rate of 13.3% (24/180) compared to 8.5% (11/130) in female macaques. This finding was consistent with another study, which reported infection rates of 2.2% (10/446) in male long-tailed macaques and 2% (4/203) in females [17]. It remains unclear whether sex also affects parasite density as there was no significant association between *Plasmodium* spp. infection and the sex of macaques after adjusting for location. While long-tailed macaques are natural hosts, differences in infection prevalence between sexes may result from biological or behavioral factors, such as immune response, activity levels, or social interactions. However, there is no clear evidence suggesting mosquito preference for either sex. Notably, this study focused on detecting the presence of infection using PCR and did not quantify parasite density. Future research incorporating qPCR, microscopy, and studies on mosquito feeding behavior and host immunity could provide deeper insights into the role of sex in malaria transmission dynamics.

In Thailand, between 2007 and 2018, malaria cases caused by simian malaria parasites were confirmed in humans in Narathiwat, Chanthaburi, Ubon Ratchathani, Yala, and Tak provinces, with *P. knowlesi* accounting for 30 cases, *P. cynomolgi* for 21 cases, *P. inui* for 19 cases, and *P. fieldi* for 3 cases [38]. More recently, data from October 2021 to March 2022 indicated a significant rise in human malaria cases caused by *P. knowlesi*, with over 70 cases reported in Ranong, Songkhla, and Trat provinces [39]. In response, the Thai government and public health authorities implemented preventive measures, including public health advisories encouraging individuals in high-risk areas to avoid getting bitten by mosquitoes and to minimize contact with macaques [39, 40].

Although *P. cynomolgi*, *P. inui*, and *P. fieldi* have been less frequently reported in humans in Thailand, other studies have demonstrated their ability to infect humans [38, 41]. Notably, most patients with simian malaria exhibited mixed-species infections, accounting for 85.29% (58/68) of cases, with the majority coinfected with *P. vivax* [38]. These findings underscore the need for continued vigilance, not only against *P. knowlesi* but also in monitoring for the emergence of other simian malaria parasites that could pose a threat to public health.

Furthermore, studies have suggested that certain *Anopheles* species acted as vectors for simian malaria. For example, the *Anopheles gambiae* complex (a group of closely related species found in Africa) includes *Anopheles gambiae s.s.*, *Anopheles coluzzii*, and *Anopheles arabiensis* that are considered the primary vectors of human malaria in the region [42, 43]. Similarly, *Anopheles stephensi* serves as a major vector in the Indian subcontinent and parts of the Middle East [44]. In the Amazon region, *Anopheles darlingi* was identified as the primary malaria vector [45]. In Southeast Asia, the *Anopheles dirus* complex consists of species that were major malaria vectors in countries such as Thailand and Myanmar [46]. However, there is still only limited research on the vectors responsible for transmitting simian malaria in Thailand. Consequently, further studies should focus on *Anopheles* species to identify which vectors are involved in transmitting each simian *Plasmodium* species and to assess regional variations in transmission patterns. A comprehensive understanding of mosquito vector diversity is crucial for evaluating malaria transmission risks, as different *Anopheles* species may vary in their ability to host and spread specific *Plasmodium* species. Additionally, investigating emerging mosquito species and their potential role in zoonotic transmission could provide valuable insights into the evolving epidemiology of malaria. The findings from the current research are expected to contribute to the development of public health strategies aimed at controlling malaria transmission among both human and macaque populations in Thailand.

## 5. Conclusion

This study confirmed the detection of three zoonotic *Plasmodium* species, namely, *P. knowlesi*, *P. cynomolgi*, and *P. inui*, in long-tailed macaques from four provinces in Southern Thailand. Among these, *P. inui* was the most prevalent. The identification of these parasites is of major public health importance in Thailand, particularly because *P. knowlesi* is capable of infecting humans and has been associated with both morbidity and mortality. Therefore, it is critical to mitigate the risk of further spread in Thailand by raising awareness about the dangers of these parasites among local communities and implementing effective strategies to control and prevent their transmission.

## Figures and Tables

**Table 1:** Oligonucleotide sequences used for *Plasmodium* spp. detection.

Primer	Oligonucleotide sequence (5′–3′)	Target gene	Amplicon size (bp)
Genus-specific primers			
rPLU6	TTAAAATTGTTGCAGTTAAAACG	18S rRNA	1100
rPLU5	CCTGTTGTTGCCTTAAACTTC		
rPLU3	TTTTTATAAGGATAACTACGGAAAAGCTGT		240
rPLU4	TACCCGTCATAGCCATGTTAGGCCAATACC		

Genus-specific primers			
PlasmoM_N1F	ATGGCCGTTTTTAGTTCGTG	18S rRNA	—
PlasmoM_N1R	TTGTGTTAGACACACATCGTTCC		
PlasmoM_N2F	GTTAATTCCGATAACGAACGAGA		233–298
PlasmoM_N1R	TTGTGTTAGACACACATCGTTCC		

**Table 2:** Prevalence of *Plasmodium* spp. infections by species in Southern Thailand, stratified by sampling location and sex of long-tailed macaques.

Variable	No. of animals	Positive *N* (%)			
		*P. inui*	*P. knowlesi*	*P. cynomolgi*	Total
Location by province					
Phetchaburi	71	1 (1.4)	0	0	1 (1.4)
Satun	109	14 (12.8)	0	1 (0.9)	15 (13.8)
Phang Nga	30	1 (3.3)	0	0	1 (3.3)
Surat Thani	100	13 (13)	3 (3)	2 (2)	18 (18)
Sex					
Female	130	8 (6.1)	3 (2.3)	0	11 (8.5)
Male	180	21 (11.5)	0	3 (1.7)	24 (13.3)
Total	310	29 (9.3)	3 (0.9)	3 (0.9)	35 (11.3)

**Table 3:** Details of positive samples (host sex, identified *Plasmodium* species, and GenBank accession numbers).

ID	Sex	Genus-specific primer		Genus-specific primer	
		Species	Accession no.	Species	Accession no.
Phetchaburi					
PBI 57	F	—	—	Pi	PQ685654
Satun					
STN 8	M	Pi	PQ559857	—	
STN 12	M	Pi	PQ559858	—	
STN 16	M	Pi	PQ559859	—	
STN 20	F	Pi	PQ559860	—	
STN 44	M	—	—	Pi	PQ678686
STN 46	M	Pi	PQ559861	Pi	PQ678687
STN 47	M	Pi	PQ559862	Pi	PQ685653
STN 67	F	Pi	PQ559863	—	
STN 68	F	Pi	PQ559864	Pi	PQ678688
STN 69	M	Pi	PQ559865	—	
STN 71	M	Pi	PQ559866	—	
STN 82	M	Pi	PQ559867	Pi	PQ678689
STN 86	F	—	—	Pi	PQ678690
STN 87	F	—	—	Pi	PQ678691
STN 89	M	—	—	Pc	PQ678667
Phang Nga				—	
PNA 20	F	Pi	PQ559856	Pi	PQ678672
Surat Thani				—	
SNI 8	M	—	—	Pi	PQ678673
SNI 12	M	—	—	Pi	PQ678674
SNI 14	M	—	—	Pi	PQ678675
SNI 16	M	—	—	Pi	PQ678676
SNI 20	M	—	—	Pi	PQ678677
SNI 21	M	—	—	Pi	PQ678678
SNI 24	M	—	—	Pi	PQ678679
SNI 33	F	—	—	Pk	PQ678669
SNI 56	F	—	—	Pk	PQ678670
SNI 58	M	—	—	Pi	PQ678680
SNI 59	M	—	—	Pc	PQ678665
SNI 63	F	—	—	Pi	PQ678681
SNI 72	F	—	—	Pk	PQ678671
SNI 75	M	—	—	Pc	PQ678666
SNI 77	M	—	—	Pi	PQ678682
SNI 80	M	—	—	Pi	PQ678683
SNI 89	M	—	—	Pi	PQ678684
SNI 98	M	—	—	Pi	PQ678685

Abbreviations: F = female, M = male, Pc = *Plasmodium cynomolgi*, Pi = *Plasmodium inui*, and Pk = *Plasmodium knowlesi*.

**Table 4:** Prevalence and odds ratios of *Plasmodium* spp. infections in long-tailed macaques from Southern Thailand.

Variable	No. of animals	% of positive samples (*N*)	95% CI of proportion	OR	95% CI of OR	*p* value
Sex						
Female	130	8.5 (11/130)	4.3–14.6	1	Reference	
Male	180	13.3 (24/180)	8.7–19.2	1.43	0.66–3.10	0.367
Location						
Phetchaburi	71	1.4 (1/71)	0–7.6	1	Reference	
Satun	109	13.8 (15/109)	7.9–21.7	11.06	1.43–85.80	0.021^∗^
Phang Nga	30	3.3 (1/30)	0.1–17.2	2.46	0.15–40.77	0.529
Surat Thani	100	18 (18/100)	11.0–26.9	14.61	1.90–112.57	0.010^∗^
Total	310	11.3 (35/310)	8.0–15.3			

Abbreviations: CI = confidence interval, OR = odds ratio.

^∗^Denotes statistical significance (*p* < 0.05).

## Data Availability

The data that support the findings of this study are available from the corresponding author upon reasonable request.
